# Dysconnectivity in the Frontoparietal Attention Network in Schizophrenia

**DOI:** 10.3389/fpsyt.2013.00176

**Published:** 2013-12-24

**Authors:** Jonathan P. Roiser, Rebekah Wigton, James M. Kilner, Maria A. Mendez, Nicholas Hon, Karl J. Friston, Eileen M. Joyce

**Affiliations:** ^1^Institute of Cognitive Neuroscience, University College London, London, UK; ^2^Psychosis Studies, Cognition and Schizophrenia Imaging Lab, Institute of Psychiatry, King’s College London, London, UK; ^3^Wellcome Trust Centre for Neuroimaging, University College London, London, UK; ^4^Institute of Neurology, University College London, London, UK; ^5^Department of Forensic and Neurodevelopmental Sciences, Institute of Psychiatry, King’s College London, London, UK; ^6^Department of Psychology, National University of Singapore, Singapore

**Keywords:** schizophrenia, frontoparietal, magnetoencephalography, dynamic causal modeling, dysconnectivity, DCM

## Abstract

Cognitive impairment is common in patients with schizophrenia, and even those with relatively preserved function perform worse than healthy volunteers (HVs) on attentional tasks. This is consistent with the hypothesis that connectivity – in the frontoparietal network (FPN) activated during attention – is disrupted in schizophrenia. We examined attentional effects on connectivity in the FPN, in schizophrenia, using magnetoencephalography (MEG). Twenty-three HVs and 19 first-episode schizophrenia patients were scanned during a simple visual change test, known to activate the FPN, in which attention was monitored and directed with an orthogonal flicker-detection task. Dynamic causal modeling (DCM) of evoked responses was used to assess effective connectivity – and its modulation by changes in the attended stimulus dimension – in the following network: higher visual area; temporoparietal junction (TPJ); intraparietal sulcus (IPS); dorsal anterior cingulate cortex; and ventrolateral prefrontal cortex. The final MEG analysis included 18 HVs and 14 schizophrenia patients. While all participants were able to maintain attention, HVs responded slightly, but non-significantly, more accurately than schizophrenia patients. HVs, but not schizophrenia patients, exhibited greater cortical responses to attended visual changes. Bayesian model comparison revealed that a DCM with attention dependent changes in both top-down and bottom-up connections best explained responses by patients with schizophrenia, while in HVs the best model required only bottom-up changes. Quantitative comparison of connectivity estimates revealed a significant group difference in changes in the right IPS-TPJ connection: schizophrenia patients showed relative reductions in connectivity during attended stimulus changes. Crucially, this reduction predicted lower intelligence. These data are consistent with the hypothesis that functional dysconnections in the FPN contribute to cognitive impairment in schizophrenia.

## Introduction

Patients with schizophrenia exhibit reliable impairments on almost all cognitive tests (1). They exhibit particular impairments on tests that depend on attention (2, 3) and working memory (4–6). This is a pattern observed even in patients whose cognitive performance is otherwise in the normal range. Generalized cognitive impairment, indexed by intelligence quotient (IQ), precedes the onset of psychosis and is detectable as far back as infancy (7). In addition, the severity of impairment is linearly related to the risk of developing psychosis and predicts functional outcome following illness onset (7, 8). Furthermore, supporting the notion that cognitive impairment represents a core feature of the syndrome in addition to positive and negative symptoms, it has also been reported in unaffected first degree relatives of patients with schizophrenia (9–15). This suggests that the neural abnormalities underlying impaired cognition reflect the neurodevelopmental susceptibility to schizophrenia. Cognitive impairment is thus a core feature of schizophrenia that significantly impacts on the course of illness; warranting a more detailed understanding of its generalized nature and neurobiological basis.

Functional neuroimaging has highlighted a core network of prefrontal and parietal regions that is activated during the performance of a variety of disparate cognitive tasks. For example, this frontoparietal network (FPN) is activated in tasks ranging from set-shifting (16) to feature selection (17) and object orientation (18) as well as recognition memory (19), working memory (20) and attention [for reviews see Ref. (21–23)]. One hypothesis is that the FPN serves as a “multiple demand system” which undertakes the information processing requirements common to different cognitive tasks, for example by updating and maintaining changes to attended stimuli (24). This system is supported by various attention networks such as the orientation network (25, 26) as well as global connectivity within this network (27). Furthermore, the development, and thus differences, of these specific attention systems – particularly in the FPN – are driven by interactions between cognition and our environment (28). This is further supported by the findings of an fMRI study by Hon et al. (29) using a task in which subjects were instructed to attend to a subset of visual stimuli and press a button when they detected a brief “flicker.” This task robustly activated the FPN simply when attended visual stimuli changed, independent of any flickers or responses. Importantly, interposed unattended stimulus changes did not elicit FPN activation, thus implicating involvement of FPN specifically in task-relevant information processing.

The “multiple demand” hypothesis predicts that dysfunction of the FPN will adversely impact on the performance of a wide range of cognitive tasks. In schizophrenia, fMRI studies have shown that nodes in this network respond abnormally across different tasks such as attention, executive control, and working memory (19, 30, 31). However, no studies in schizophrenia have examined FPN integrity in the context of a task that recruits a fundamental attentional process – a process that is essential for the performance of many higher order tasks but which itself requires “minimal decision and control” (29, 32). We therefore investigated FPN function in schizophrenia using the task described by Hon et al. (29).

Regarding possible pathophysiological mechanisms underlying schizophrenia, the “dysconnection” hypothesis is one of the most influential (33–35). The central tenet of this model is that the symptoms observed in schizophrenia arise from abnormal regulation of *N*-methyl-d-aspartate (NMDA) receptor-dependent synaptic efficacy by modulatory transmitters, such as dopamine or acetylcholine (35). This is proposed to result in abnormal integration of neural processes, which can be measured in terms of effective connectivity, or the influence that one neural system has over another (36). Using dynamic causal modeling (DCM) – which estimates effective connectivity – prior studies have found that aberrant perceptual processes in schizophrenia are associated with such dysconnectivity (37, 38). These differences in effective connectivity are thought to reflect aberrant NMDA receptor-dependent regulation of synaptic efficacy; similar dysfunction may also occur in the FPN, resulting in dysfunctional processing in the multiple demand system and thus impairment on several different types of cognitive tests. Furthermore, disrupted connectivity assessed using DCM has also been reported during tasks that examined working memory and verbal fluency in individuals at risk for psychosis (39–41). However, most previous studies assessing differences in connectivity employed functional magnetic resonance imaging (fMRI), which precludes the use of detailed and neurophysiological plausible (neural mass) models, due to its low temporal resolution.

In this study we therefore measured neural responses in patients with schizophrenia and healthy volunteers (HVs) with magnetoencephalography (MEG) during a simple visual change task known to engage the FPN (29); we assessed effective connectivity in the FPN with DCM. MEG, in particular, provides more temporally precise data than fMRI. This detailed time course information enables the efficient estimation of much more realistic and detailed neural mass models – particularly models with different types of connection (i.e., forward; backward; and lateral) among distinct neuronal populations. Cortical areas previously reported to be activated by the task employed (using fMRI) were used as regions of interest (ROIs) when specifying the different neural network models for DCM [see Figure 2 and Table 2 of Ref. (29)]. DCM uses these co-ordinates as spatial priors when specifying the location of the electromagnetic sources that generate sensor-space responses. With these models, we were able to estimate how effective connectivity between nodes in the FPN – and its modulation by attended visual changes – differs between patients with schizophrenia and HVs. We predicted that patients with schizophrenia would show abnormal effective connectivity (and its modulation by attention), as reflected in the coupling estimates from DCM, and – crucially – that these would predict cognitive impairment (i.e., low IQ).

## Materials and Methods

### Participants

Nineteen patients with schizophrenia were recruited from a longitudinal study of cognitive impairment in first-episode psychosis (6). The diagnosis was ascertained using a structured interview, the diagnostic module of the Diagnostic Interview for Psychosis (42), which includes items from the Operational Criteria Checklist for Psychosis (43) and the World Health Organisation Schedules for Clinical Assessment in Neuropsychiatry (44). Diagnoses were made at study entry and reviewed 1 year later. Twenty-three HVs were recruited via advertisement.

Exclusion criteria for all participants included: medical disorders likely to cause cognitive impairment; IQ less than 70; left-handedness; and recent illicit substance use. Additional exclusion criteria for HVs included: past or present psychiatric or neurological disorders; and any first degree relatives with a psychotic illness. All HVs were screened for Axis-I psychopathology using the Mini International Neuropsychiatric Interview (45). One healthy volunteer was excluded because he had a brother with psychosis; another was excluded for being left-handed. Three HVs and two patients with schizophrenia were excluded because we were unable to identify clear visual evoked responses. Two patients with schizophrenia were excluded due to failure to follow task instructions. One patient with schizophrenia was excluded because her diagnosis was subsequently changed to depression with psychosis. After exclusions, the final MEG analysis included 18 HVs (9 male, 9 female) and 14 patients with schizophrenia (10 male, 4 female), all right-handed.

Demographic and clinical data are presented in Table 1. The groups were similar in terms of age, premorbid IQ [estimated using the National Reading Test: (46)], and current IQ [estimated using the Wechsler scale of adult intelligence (WAIS)-III] (47). All patients but one were taking antipsychotic medication; four were also taking antidepressant medication; and one was only taking antidepressant medication. None of the HVs were taking any psychotropic medication. Symptom severity scores for the patients were measured using the Scale for Assessment of Negative Symptoms (SANS) (48) and the Scale for Assessment of Positive Symptoms (SAPS) (49). All participants provided written informed consent before the start of the experiment and were compensated for participation. The study was approved by Ealing and West London Local Research Ethics Committee.

**Table 1:** **Demographic and clinical sample characteristics**.

	Patients with schizophrenia (*N* = 14) [mean (SD)]	Healthy volunteers (*N* = 18) [mean (SD)]	Statistics
Age (years)	25.21 (4.23)	24.11 (6.00)	*t*(30) = 0.584, *P * > 0.1
WAIS-III Byler IQ	101.43 (19.23)	106.83 (15.22)	*t*(30) = 0.888, *P * > 0.1
NART verbal IQ	101.57 (7.94)	99.33 (9.63)	*t*(30) = 0.703, *P * > 0.1
Gender	10M, 4F	9M, 9F	χ^2^(1) = 1.50, *P* > 0.1
Age at onset (years)	21.43 (4.85)	–	
Duration of illness (years)	3.79 (1.37)	–	
CPZ equivalents	247.62 (143.80)	–	
SANS global ratings		–
Affective flattening or blunting	6.86 (8.47)	–	
Alogia	5.80 (6.76)	–	
Anhedonia-asociality	3.14 (4.31)	–	
Attention	0.21 (0.80)	–	
Overall	19.1 (21.9)	–	
SAPS global ratings		–
Hallucinations	3.79 (6.13)	–	
Delusions	4.64 (5.73)	–	
Bizarre behavior	0.29 (1.07)	–	
Positive formal thought disorder	0.86 (2.48)	–	
Overall	9.6 (14.0)	–	

*IQ, intelligence quotient; M, male; F, female*.

*WAIS, Wechsler adult intelligence scale; NART, national adult reading test; CPZ, chlorpromazine equivalent (72, 73); SANS, scale for assessment of negative symptoms; SAPS, scale for assessment of positive symptoms*.

### Cognitive activation paradigm

**Figure 1:**
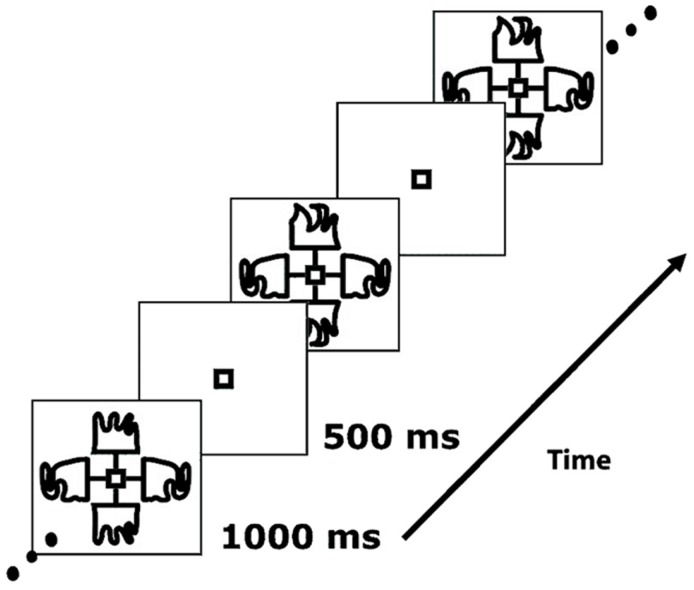
**Example of a stimulus sequence used in the task (29)**.

The paradigm was presented as described in Hon et al. (29), with the exception that all stimuli were black and white. Participants were presented with a series of complex, nonsense shapes, similar to those shown in Figure 1. The images were displayed to the left, right, top and bottom of a central box. Each stimulus on either the vertical or horizontal axis was a mirror reflection of the opposite image. The shapes were hand drawn to ensure that they did not resemble any familiar patterns or objects. Each stimulus was presented for a period of 1000 ms before an off period of 500 ms, during which only the central box appeared. All the images and lines appeared and disappeared at the same time. This task comprised two blocks, each consisting of 180 trials, and lasted four and a half minutes per block. For each trial, either: (1) the shapes on the horizontal axis changed, in relation to the previous trial but the shapes on the vertical axis remained the same (60 occurrences); or (2) the shapes on the vertical axis changed and the shapes on the horizontal axis remained the same (60 occurrences); or (3) no shape change occurred on either axis (60 occurrences). On one-third of trials (20 in each condition) either the horizontal or the vertical axis flickered briefly. Trial types were presented in a random order. In order to maintain stimulus novelty, no shape was reused in each block.

This task was chosen to be sufficiently straightforward so that patients with schizophrenia would be able to perform it to a high level of accuracy. Participants were instructed to maintain fixation on the box in the center of the screen, which was confirmed using concurrent eye-tracking. During each run, participants were instructed to watch either the horizontal axis or the vertical axis, and press a button on an MEG-compatible button-box with their right index finger only when a flicker occurred on the axis they were attending to. The order was counterbalanced across participants. Two runs were conducted for each participant, each lasting 4.5 min.

### Magnetoencephalography

#### MEG data acquisition

Magnetoencephalography data were recorded using 275 first order axial gradiometers with the Omega275 CTF MEG system (VSMmedtech, Vancouver, Canada) at a 480 Hz sampling frequency. Data were recorded in a magnetically shielded room. Each participant’s head was supported by a padded headrest to restrict head movement during recording. To monitor head motion, sensors were placed near the ears and nose. None of the participants included in the final analysis exceeded a threshold of 5 mm for head movement within a single run. Eye-tracking was also used to ensure that all participants attended continually to the visual stimuli.

#### MEG data analysis

Data analysis was performed using Statistical Parametric Mapping 12 (SPM12) (Wellcome Trust Centre for Neuroimaging, London, UK; www.fil.ion.ucl.ac.uk/spm) in MATLAB 12.1 (MathWorks Inc., Sherbon, MA, USA). Blocks with attended flicker-detection (hit) rates lower than 60% or incorrect response (false alarm) rates greater than 15%, were excluded from the analysis. The remaining data were epoched with a peristimulus window from −100 to 1300 ms. Data were processed using artifact detection routines to identify and exclude eye blinks or movement before bandpass filtering between 0.5 and 16 Hz. The data were then down sampled to 200 Hz and baseline corrected using the −100–0 ms period. One bad MEG channel was excluded from the analysis across all participants.

All flicker and false alarm trials (i.e., if the participant made a response when the attended axis had not flickered) were removed from further analysis. Robust averages were calculated for each event type (attended change, unattended change and no change) across all valid trials for each session – note that no trial included in the analysis featured a flicker stimulus or any response by the participant. Grand average responses were created for each participant for all sessions that met the performance criteria specified above. These average responses were interpolated into images and visually inspected for artifacts and the presence of visual evoked fields (VEFs). Data were excluded if a clear VEF could not be identified over occipital sensors. From these grand average images, contrast images were created for the following comparisons: (attended change minus unattended change); (attended change minus no change); and (unattended change minus no change). A smoothing kernel of (2 mm × 2 mm × 2 mm) was applied to each contrast image.

These sensor-space contrast images were combined at the group-level for random-effects analysis, which was performed across all sensors and all time points. For group comparisons, significant clusters were defined as those surviving an uncorrected voxel-level threshold of *P * < 0.001 with a cluster-level threshold corrected across the whole of sensor-space and peristimulus time, controlling the family-wise error (FWE) rate at *P * < 0.05. For within-group contrasts, which produced extensive activation in HVs, we used an uncorrected voxel-level threshold of *P * < 0.0005, again controlling the FWE rate at *P * < 0.05 at the cluster-level.

#### Dynamic causal modeling

Dynamic causal modeling was applied to assess effective connectivity. DCM uses the concept of effective connectivity, or the influence that one neural system has over another, to create a model of coupled neuronal populations that is used to explain evoked responses (50). The parameters (effective connectivity and other synaptic constants) are optimized by fitting responses generated by the model – in response to stimuli – to observed responses using standard Bayesian model inversion techniques (51). In addition, the evidence for a particular model (irrespective of the particular parameters) is evaluated in terms of model evidence through Bayesian model comparison (BMC) (52, 53). Crucially, DCM estimates not just the effective connectivity or coupling between sources of electromagnetic responses but also sets of experimental changes in coupling. This allows one to use BMC to assess the evidence for context dependent changes in connectivity – such as changes induced by the nature of the stimulus (attended versus unattended). This method incorporates an exceedance probability to determine the best fitting model or the likelihood that one model fit the data better than any of the other models.

**Figure 2:**
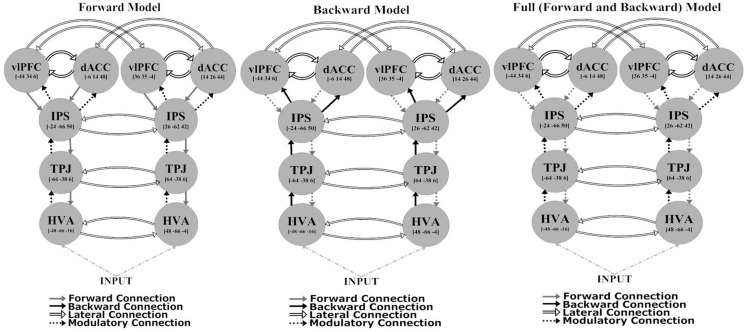
**Models used for the dynamic causal modeling analysis**.

The network architecture used for the DCM comprised sources that were previously identified as being activated in an fMRI study employing the same task (29). These sources were consistent with the most robust responses in the (attended change minus unattended change) analysis of sensor-space responses [see Figure 2 and Table 2 of Ref. (29)]. They included: higher visual area (HVA) ([48, −66, −4] and [−48, −66 −16]); temporoparietal junction (TPJ) ([64, −38, 6] and [−64, −38, 6]); intraparietal sulcus (IPS) ([26, −62, 42] and [−24, −66, 50]); ventrolateral prefrontal cortex (vlPFC) ([36, 35, −4] and [−44, 34, 6]); and dorsal anterior cingulate (dACC) ([14, 26, 44] and [−6, 14, 48]). All models had a fixed model architecture within each hemisphere (both forward and backward connections) as follows: between HVA and TPJ; between TPJ and IPS; between IPS and dACC; and between IPS and vlPFC. Furthermore, all models had fixed lateral connections between vlPFC and dACC, and inter-hemispherically between all corresponding regions (i.e., between left and right HVA, TPJ, IPS, vlPFC, and dACC) (see Figure 2). Using the above co-ordinates as spatial location priors, three DCMs were constructed, varying in relation to modulatory effects – the modulation attributable to a stimulus change on the attended dimension or axis – on forward connections (reflecting bottom-up effects), on backward connections (reflecting top-down effects), or on both forward and backward connections (see Figure 2). This approach is consistent with prior studies using DCM for MEG (38, 54). All models had driving inputs into the HVA, modeling subcortical visual input.

Each DCM was estimated at the subject level, but BMC was conducted at the group-level, in each group separately, to determine the most parsimonious explanation for the data in terms of attentional modulation of connectivity (models with the most evidence represent an accurate explanation of observed data with minimal complexity). To make quantitative inferences about differences in connection strengths, effective connectivity, and its modulation were compared using classical statistics at the group-level. Fixed and modulatory coupling estimate parameters (indicating the strength of each fixed connection and its modulation by attended visual change, respectively) were computed using random-effects Bayesian model averaging (BMA) (55), and submitted to analysis in SPSS 17 (SPSS Inc., Chicago, IL, USA). After BMA, we obtained connectivity parameters for all fixed and modulatory connections (only forward and backward) for each hemisphere as well as the intrahemispheric connections: total 46 parameters. However, we did not examine any of the intrahemispheric connections so this left a total of 36 connections that were examined for group differences.

Group differences were assessed with *t*-tests and relationships with demographic, clinical, and cognitive variables with Pearson’s *r* correlation coefficients. These tests were not corrected for multiple comparisons. Since this was an exploratory analysis, and because our primary inference related to the BMC, an alpha level of 0.05 was adopted for all group comparisons. We refer to trend significance at a threshold of 0.05 < *P * < 0.1. However, to reduce the number of correlations performed, only those coupling parameters that differed between the groups at least at a trend level were entered into correlation analyses.

## Results

### Behavioral performance

Patients with schizophrenia responded slightly, but non-significantly, less accurately than HVs, with a lower hit rate (Table 2). There were no significant differences between the groups in terms of reaction times, false alarms or *d*′. Concurrent eye-tracking confirmed that all participants attended to the visual stimuli throughout the task.

**Table 2:** **Behavioral data from the visual change task**.

	Patients with schizophrenia, mean (SD)	Healthy volunteers, mean (SD)	Statistics
Correct hits (flickers detected) (%)	78.69 (9.90)	85.16 (8.96)	*t*(30) = 1.943, *P * = 0.061
False alarms (inappropriate responses) (%)	2.38 (2.24)	2.87 (2.90)	*t*(30) = 0.521, *P * = 0.606
Correct hit reaction time (ms)	499.02 (91.85)	466.51 (53.24)	*t*(30) = 1.248, *P * = 0.218
*d*′	2.88 (0.46)	3.21 (0.75)	*t*(30) = 1.459, *P * = 0.155

*SD, standard deviation; ms, milliseconds*.

### MEG sensor-space responses

**Figure 3:**
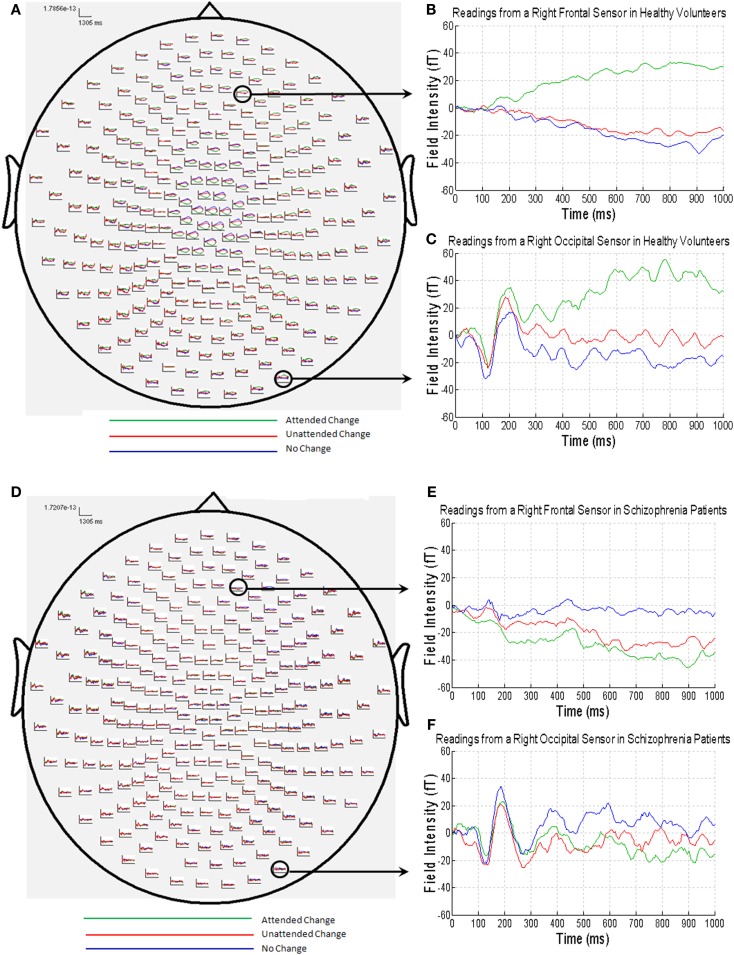
**(A)** MEG responses averaged over all of the healthy volunteers with attended change trials in green, unattended change trials in red and no change trials in blue; **(B)** MEG data from a right frontal sensor; **(C)** MEG data from a right occipital sensor, showing the visual evoked field in response to stimulus presentation; **(D)** MEG responses averaged over all of the patients with schizophrenia (colors as above); **(E)** MEG data from a right frontal sensor; **(F)** MEG data from a right occipital sensor, showing the visual evoked field in response to stimulus presentation.

The MEG grand average image in the healthy controls (Figures 3A–C) showed a consistent and sustained deviation in cortical responses between attended and unattended stimulus changes from approximately 200 ms after stimulus onset, primarily in right lateral frontal sensors. This increase was evident for almost 1 s. This deviation becomes statistically significant (cluster-level correction) at approximately 375 ms over right frontal sensors [peak at 395 ms: *t*(17) = 4.87, *P*_FWE_* * = 0.039]. The response at this peristimulus time is consistent with the P300 response often observed in oddball paradigms (56, 57), of which our task is a variant (with an attentional manipulation). Similar effects were observed later in peristimulus time over the same sensors, and over more anterior frontal sensors (see Table 3).

**Table 3:** **Results for contrast images for MEG sensor-space responses**.

Approximate location	Time (ms)	*k*	*Z*-score	*P* (cluster corrected)
Attended > unattended change for healthy volunteers^a^				
Right occipital/temporal	75	188	4.44	0.280
Mid frontal	685	284	4.07	0.091
Left parietal	755	173	4.02	0.333
Mid frontal	895	2066	3.98	<0.001*
Right frontal	715	380	3.90	0.031*
Mid frontal	905	155	3.88	0.409
Right frontal	395	359	3.80	0.039*
Right frontal	800	545	3.74	0.006*
Right frontal	540	264	3.73	0.115
Left frontal	315	137	3.69	0.498
Mid frontal	555	50	3.67	0.960
Mid frontal	710	61	3.59	0.923
Unattended > attended change for healthy volunteers^a^				
No clusters survived threshold
Attended > unattended change for schizophrenia patients^a^				
No clusters survived threshold
Unattended > attended change for schizophrenia patients^a^				
No clusters survived threshold
Attended > unattended change for healthy volunteers > schizophrenia patients^b^				
Left parietal	755	14	3.45	0.999
Right frontal	260	22	3.39	0.999
Right frontal	375	14	3.25	0.999
Right frontal	320	12	3.18	0.999
Unattended > attended change for healthy volunteers > schizophrenia patients^b^				
No clusters survived threshold
Attended > unattended change for schizophrenia patients > healthy volunteers^b^				
No clusters survived threshold
Unattended > attended change for schizophrenia patients > healthy volunteers^b^				
No clusters survived threshold				

*^a^Analyses within groups thresholded at *P * < 0.0005, cluster size 40 voxels*.

*^b^Analyses between groups thresholded at *P * < 0.001, cluster size 10 voxels*.

*P  < 0.05 FWE whole-brain cluster-level corrected.

**k* = number of contiguous voxels*.

By contrast, the MEG responses from the patients with schizophrenia showed no difference between the attended change and unattended change conditions (Figures 3D–F). This finding was paralleled in the statistical analyses, where no differences in sensor-space responses were observed between any of the conditions, even when the threshold was lowered to *P * < 0.001 (uncorrected), extent threshold 10 voxels. However, patients with schizophrenia did, nonetheless, show a clear VEF across all conditions (Figure 3F), suggesting that they did engage with the task and have detectable electromagnetic responses.

Although patients with schizophrenia exhibited diminished cortical responses to attended visual change at frontal sensors in comparison to the controls (Figures 3B,E), no group differences survived stringent correction for multiple comparisons across the whole of sensor-space and peristimulus time at any time point (Table 3). However, at a more liberal threshold of *P * < 0.001 (uncorrected), extent threshold 10 voxels, diminished responses in the schizophrenia patients were detected over right frontal sensors at 260 ms [*t*(30) = 3.78, *P * < 0.001, uncorrected], 320 ms [*t*(30) = 3.50, *P * < 0.001, uncorrected], 360 ms [*t*(30) = 3.55, *P * < 0.001, uncorrected], and 375 ms [*t*(30) = 3.60, *P * < 0.001, uncorrected], and over left parietal sensors 755 ms [*t*(30) = 3.85, *P * < 0.001, uncorrected] (see Table 3). We report these results descriptively, noting that they require replication.

Neither group showed any activation in the (unattended change minus no change) contrast at a threshold of *P * < 0.0005 uncorrected, extent threshold 40 voxels. The (attended change minus no change) contrast produced similar results to the (attended change minus unattended change) contrast.

### Dynamic causal modeling

#### Bayesian model comparison

**Figure 4:**
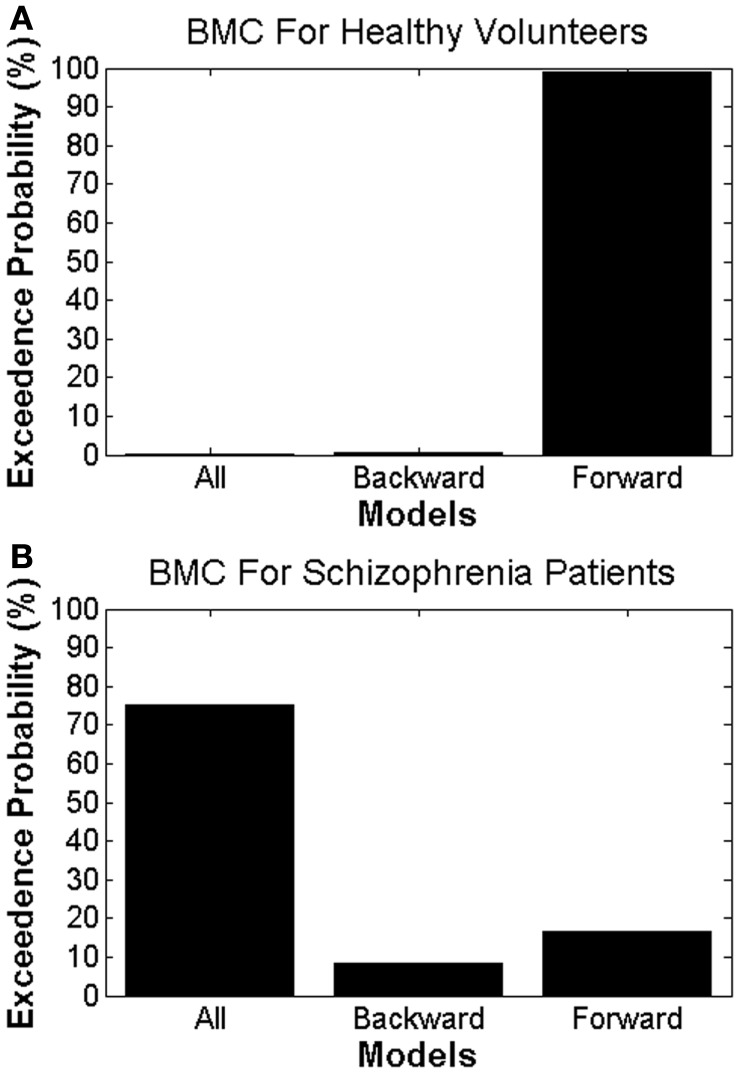
**Bayesian model comparison results for (A) healthy volunteers and (B) patients with schizophrenia, showing the exceedance probabilities for each DCM model**.

Using BMC, we were able to determine which model provided the most parsimonious explanation for the effects of attended stimulus changes in both groups. Different DCMs provided the best explanation in patients with schizophrenia and HVs, providing *prima facie* evidence for dysconnectivity during attentional processing in schizophrenia. In HVs, there was greatest evidence for the model with only forward modulations, with an exceedance probability of 99% over other models. In patients with schizophrenia there was greatest evidence for the model with both forward and backward modulations, with an exceedance probability of 75% over the other models (Figure 4).

#### Quantitative connectivity estimates

To understand better why the groups differed in terms of the most parsimonious architecture, we quantified the connectivity and its attentional modulation, using BMA (Table 4). This effectively weights the coupling estimates, under each model, according to the probability of that model and accommodates uncertainty about the underlying architectures.

**Table 4:** **Connectivity estimates from Bayesian model averaging**.

	Group difference statistic	Schizophrenia patients, mean (SD)	Healthy volunteers, mean (SD)
Fixed connections
Forward
Right HVA to TPJ	*t*(30) = 0.687, *P * = 0.498	−0.001 (0.130)	0.037 (0.178)
Left HVA to TPJ	*t*(30) = 0.152, *P * = 0.880	0.010 (0.179)	0.029 (0.172)
Right TPJ to IPS	*t*(30) = 0.854, *P * = 0.400	0.030 (0.232)	0.094 (0.192)
Left TPJ to IPS	*t*(30) = 0.876, *P * = 0.388	0.036 (0.131)	0.090 (0.204)
Right IPS to dACC	*t*(30) = 1.678, *P * = 0.104	0.065 (0.162)	−0.019 (0.119)
Left IPS to dACC	*t*(30) = 2.019, *P * = 0.052*	0.062 (0.188)	0.219 (0.240)
Right IPS to vlPFC	*t*(30) = 0.932, *P * = 0.359	0.118 (0.180)	0.061 (0.163)
Left IPS to vlPFC	*t*(30) = 0.758, *P * = 0.455	0.085 (0.170)	0.034 (0.206)
Backward
Right TPJ to HVA	*t*(30) = 1.352, *P * = 0.187	0.010 (0.208)	−0.097 (0.233)
Left TPJ to HVA	*t*(30) = 1.049 *P * = 0.302	0.038 (0.192)	−0.029 (0.172)
Right IPS to TPJ	*t*(30) = 0.912, *P * = 0.514	0.000 (0.109)	−0.027 (0.125)
Left IPS to TPJ	*t*(30) = 0.300, *P * = 0.766	0.018 (0.172)	0.003 (0.115)
Right dACC to IPS	*t*(30) = 0.946, *P * = 0.351	−0.035 (0.124)	0.030 (0.232)
Left dACC to IPS	*t*(30) = 0.254, *P * = 0.801	0.102 (0.307)	0.078 (0.216)
Right vlPFC to IPS	*t*(30) = 1.666, *P * = 0.106	0.104 (0.182)	0.004 (0.160)
Left vlPFC to IPS	*t*(30) = 0.271, *P* = 0.788	0.000 (0.167)	0.016 (0.168)
Lateral
Right dACC to vlPFC	*t*(30) = 0.476, *P * = 0.638	−0.031 (0.126)	−0.008 (0.136)
Left dACC to vlPFC	*t*(30) = 0.356, *P * = 0.724	−0.056 (0.156)	−0.037 (0.150)
Right vlPFC to dACC	*t*(30) = 0.425, *P * = 0.674	0.034 (0.148)	0.015 (0.106)
Left vlPFC to dACC	*t*(30) = 1.070, *P * = 0.293	0.013 (0.142)	−0.038 (0.128)
Modulatory connections
Forward
Right HVA to TPJ	*t*(30) = 0.352, *P * = 0.728	−0.043 (0.209)	−0.013 (0.264)
Left HVA to TPJ	*t*(30) = 0.725, *P * = 0.474	−0.018 (0.255)	0.041 (0.207)
Right TPJ to IPS	*t*(30) = 0.857, *P * = 0.398	0.009 (0.189)	−0.057 (0.233)
Left TPJ to IPS	*t*(30) = 1.165, *P * = 0.253	0.014 (0.254)	−0.082 (0.219)
Right IPS to dACC	*t*(30) = 1.701, *P * = 0.099*	0.019 (0.172)	−0.092 (0.192)
Left IPS to dACC	*t*(30) = 0.614, *P * = 0.544	0.025 (0.149)	0.059 (0.163)
Right IPS to vlPFC	*t*(30) = 1.236, *P * = 0.226	0.060 (0.172)	−0.015 (0.166)
Left IPS to vlPFC	*t*(30) = 0.087, *P * = 0.931	−0.044 (0.200)	−0.050 (0.204)
Backward
Right TPJ to HVA	*t*(30) = 1.445, *P * = 0.159	0.073 (0.236)	−0.030 (0.168)
Left TPJ to HVA	*t*(30) = 1.002, *P * = 0.324	−0.026 (0.254)	0.048 (0.161)
Right IPS to TPJ	*t*(30) = 2.428, *P * = 0.021**	−0.063 (0.116)	0.036 (0.115)
Left IPS to TPJ	*t*(30) = 1.269, *P * = 0.214	0.065 (0.188)	−0.010 (0.146)
Right dACC to IPS	*t*(30) = 0.567, *P * = 0.575	0.006 (0.178)	−0.023 (0.115)
Left dACC to IPS	*t*(30) = 1.527, *P * = 0.137	−0.017 (0.144)	0.069 (0.169)
Right vlPFC to IPS	*t*(30) = 0.174, *P * = 0.863	0.042 (0.164)	0.031 (0.178)
Left vlPFC to IPS	*t*(30) = 1.133, P = 0.266	0.053 (0.167)	−0.018 (0.178)
Input
Right HVA	*t*(30) = 1.073, *P * = 0.292	−0.107 (0.207)	−0.026 (0.214)
Left HVA	*t*(30) = 0.081. *P * = 0.936	−0.063 (0.193)	−0.068 (0.168)

*HVA, higher visual area; TPJ, temporoparietal junction; IPS, intraparietal sulcus; dACC, dorsal anterior cingulate cortex; vlPFC, ventrolateral prefrontal cortex*.

*Trend toward significance (*P * < 0.1).

**P  < 0.05.

##### Fixed connections

For the fixed connections, the left IPS-dACC fixed forward connection was marginally stronger in the HVs [*t*(30) = 2.019, *P * = 0.052]. This means that HVs show a trend toward being relatively more sensitive to parietal afferents to the dACC than schizophrenia patients, irrespective of whether the stimulus change was on the attended dimension or not.

##### Modulatory connections

For the modulatory connections (i.e., altered coupling elicited by attended visual change), only the right IPS-TPJ modulation differed significantly between the groups [*t*(30) = 2.428, *P * = 0.021]. This modulator was negative in schizophrenia and slightly positive in healthy subjects. In other words, the top-down afferents from the parietal source to the TPJ were reduced in their strength in schizophrenia, relative to HVs, only when the stimulus changed in the attended dimension. Note that a negative modulation corresponds to a reduction in connectivity (because connection strengths in DCM for electromagnetic responses are always positive – targeting excitatory and inhibitory neuronal populations).

There was also a trend toward a group difference in the right IPS-dACC modulation [*t*(30) = 1.701, *P * = 0.099], with a negative modulator in HVs but a positive modulator in schizophrenia patients.

#### Correlations with demographic, clinical, and cognitive measures

**Figure 5:**
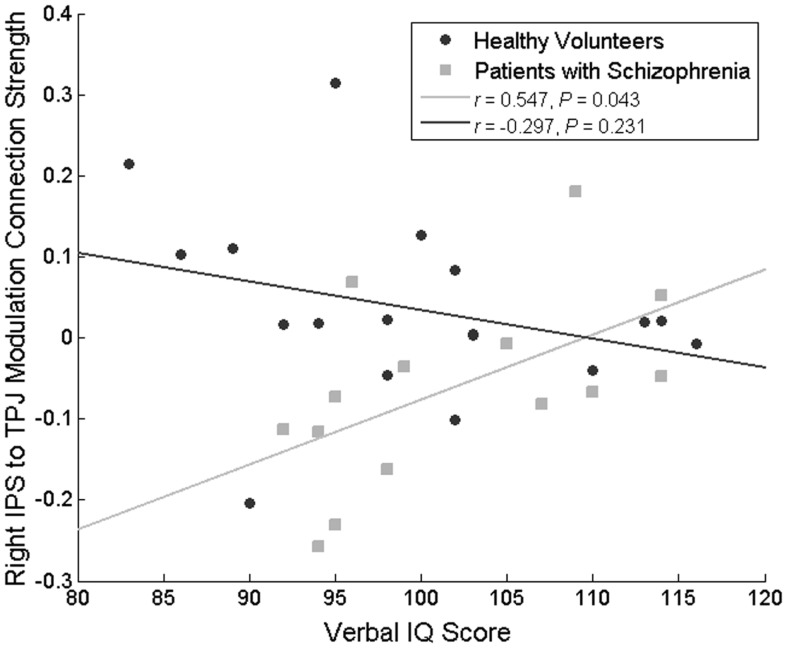
**Correlation between the attentional modulation of the IPS to TPJ backward connection and premorbid IQ**. This relationship was significant only in patients with schizophrenia.

No correlations approaching significance were identified between the left IPS-dACC fixed connection, and behavior, current IQ, premorbid IQ, age, SAPS, SANS, or chlorpromazine equivalent dose. However, changes in the IPS-TPJ connectivity correlated positively with premorbid IQ score in the patient group (*r * = 0.547, *P * = 0.043) but not in the HVs (*r * = −0.297, *P * = 0.231; difference in correlation coefficients: Fisher’s *Z* = 2.32, *P * = 0.02) (Figure 5). In other words, patients with the most (abnormal) decrease in this connection during attended stimulus change had the lowest premorbid IQ. Importantly, this modulation did not correlate significantly with the percentage of correct flicker-detections (*r * = 0.127, *P * = 0.665, in patients with schizophrenia and *r * = -0.250, *P * = 0.318 in HVs), suggesting that this difference does not simply reflect poor engagement in the task.

## Discussion

Using DCM, we found that patients with schizophrenia and HVs differ in their recruitment of the FPN during the processing of salient (attended) visual changes. Both inference about the architectures – and the connectivity of those architectures – pointed to an abnormality in top-down modulation of stimulus evoked responses, when stimulus changes were in the attended dimension or axis. In patients with schizophrenia the winning model featured modulation of both backwards (top-down) and forwards (bottom-up) connections. Specifically, patients with schizophrenia showed a relative failure (decreased connection strength) of the backward IPS-TPJ connection when processing attended visual changes.

Interestingly, this quantitative reduction was associated with lower premorbid IQ in schizophrenia patients only. In previous studies we showed that lower premorbid and current IQ (markers of general cognitive ability) at illness onset predicted poorer social function after 4 years (8). Another study also found that lower IQ and higher psychotic symptoms correlated with a loss in gray matter tissue (58). Previous studies also found that global connectivity within the FPN correlated negatively with IQ (27). We have also reported that in patients with schizophrenia, but not healthy controls, lower premorbid and current IQ was related to reduced frontotemporal cortical area and predicted progressive parietal cortical thinning over the following 3 years (59, Gutiérrez-Galve, unpublished observations). Although in the present study we did not find relationships with current IQ, taken together our findings support the hypothesis that FPN dysfunction is the basis of generalized cognitive impairment in schizophrenia (60).

Differences in connectivity were observed between the groups in several parts of the FPN, all involving parietal cortex connections. The left forward (bottom-up) IPS-dACC fixed connection showed a trend toward being stronger in HVs. This would be consistent with a relatively inefficient transmission of information from parietal to frontal regions in schizophrenia, and could explain the lack of frontal cortical activity elicited by attended visual changes in patients. We also identified a trend difference in the modulation of the right IPS-dACC forward (bottom-up) connection, which was negative only in HVs. However, we note that neither of these differences achieved statistical significance. The only effect to differ significantly between the groups was in the right IPS-TPJ backward (top-down) connection: this connection was lower in patients with schizophrenia.

These results were consistent with the sensor-space results which indicated reduced cortical responses to attended visual changes in patients with schizophrenia, consistent with prior reports of attenuated P300 responses in this group (61). Our DCM suggests that these attenuated responses are caused by selective differences in coupling between the TPJ, parietal cortex, and anterior cingulate. Importantly, the MEG results we observed are unlikely to reflect solely a lack of engagement with the task, as all patients included in the analysis could perform at a high level and, as a group, did not differ from the HVs in terms of reaction times or false alarms; although, we did observe a slight impairment in percentage correct flicker-detection. However, this measure did not correlate with any of the DCM parameters. Together, these results are consistent with the dysconnection hypothesis of schizophrenia – which proposes that aberrant cortical coupling arises due to aberrant regulation of NMDA-dependent synaptic plasticity. Within the FPN we speculate that this may lead to differences in the efficiency in which regions of the FPN are able to interact, resulting in altered effective connectivity, as assessed with MEG. Furthermore, these fit findings comfortably with recent computational accounts of dysconnectivity as a failure to adjust or contextualize the synaptic efficacy that underlies the routing of salient or precise sensory information (62).

It is interesting to interpret our results in relation to computational formulations of the dysconnection hypothesis – in particular, predictive coding models of hierarchical Bayesian inference. In these formulations, psychotic symptoms are understood in terms of the false inference that arises when the salience or precision afforded sensory information is aberrant (62–64). Formally, top-down or descending predictions in cortical hierarchies try to explain representations at lower levels by forming prediction errors at each level of the hierarchy. Ascending prediction errors are then passed forward to improve the representations at higher levels – and thereby minimize prediction error at every hierarchical level. Crucially, the influence prediction errors have on high level representations depends upon their precision (inverse variance or reliability), which itself has to be optimized (62, 65). Physiologically, precision is thought to be encoded by the postsynaptic neuromodulatory gain of superficial pyramidal cells reporting prediction error (66). Psychologically, the optimization of precision provides a simple explanation for attentional gain; in other words, the selection of ascending prediction errors that are considered salient of precise in any given context (65).

This formulation is particularly relevant here, for two reasons: first, it provides a computational account of dysconnection – that can be reduced to an abnormality of message passing during perceptual inference that rests explicitly on the aberrant modulation of synaptic efficacy (62). Second, it speaks to the important role of attentional deficits in disclosing this aberrant modulation. Although speculative, it is tempting to interpret our results from this computational perspective. The results of the DCM in HVs are entirely consistent with predictive coding; in that attention endows ascending prediction errors from lower (temporal) regions with greater precision and preferential access to higher (parietal) regions – resulting in an increase in forward effective connectivity (67). However, in schizophrenia it appears that the descending predictions – that are informed by ascending prediction errors – “fall on deaf ears,” when descending to the temporal region. This is evidenced by a reduction in the backwards connection. Although this account is somewhat heuristic, it is consistent with the fact that backward connections target superficial cortical layers that are rich in NMDA receptors – receptors that are crucial for neuromodulatory effects and play a central role in the dysconnection hypothesis (35).

Prior studies have highlighted the importance of the interaction between the IPS and the TPJ in the detection of behaviorally salient events (23). The interaction between these two areas can be split between two proposed FPN systems: the dorsal FPN, which is responsible for the orientation and maintenance of selective attention (26, 68); and the ventral FPN, which acts to direct attention to salient events (23) – and aids in the application of attentional set (69). The dorsal FPN also plays an important role in the synchronization of activity between the visual cortex and other areas of the dorsal FPN as a means of mediating top-down visual attention (69) to exert control over tasks from bottom-up sensory signals (26). It is thought that these networks are co-activated during stimulus-driven reorientation when a salient and behaviorally relevant event occurs, and is highly right-lateralized (69). Differences in integration between these systems of the FPN are evident in the connection between the IPS and TPJ; where the IPS plays a greater role in the dorsal FPN and the TPJ is more involved in the ventral FPN (23, 69). The reduction in the strength in this connection in patients with schizophrenia during attended stimulus change could be interpreted as a relative failure of functional integration between these two regions. Furthermore, a failure of this backward (top-down modulation) in attentional processing of salient events may thus explain why patients with schizophrenia recruit a more complicated connectivity architecture during the processing of visual change. Due to this connection’s role in both the dorsal and ventral FPN, we speculate this relative failure in top-down connectivity might contribute to the difficulty that patients with schizophrenia face in modifying behavior in response to salient stimuli, though this requires testing in future studies.

The groups included in this study were well matched in terms of their task performance and demographic variables. Although patients with schizophrenia made slightly fewer correct responses when detecting flickers, the false alarm rate and reaction times were similar to those of the healthy volunteer group, and the average performance in schizophrenia patients exceeded 75%. Despite performing well above chance and close to controls in being able to detect attended stimulus flickers, the activation of the FPN in schizophrenia patients to attended visual change was diminished. The implication of this finding is that the updating of attended information necessary to perform more complex tasks would be compromised due to dysconnectivity within this network, which could account for poor cognitive function more generally.

Several limitations of our study merit comment. The first is the low number of participants that we were able to include in the final analysis. This affects the statistical sensitivity (Type-II error) of our analyses. It is possible that there are other differences between the groups that we did not have sufficient sensitivity to detect. Second, the relatively preserved task performance we observed is not common to most studies of attention and working memory in schizophrenia (70), and indicates that the patients included in the present study were high-functioning. This subgroup of patients may not exhibit as extreme FPN network dysconnectivity as other groups, which would also raise the chance of Type-II error, and means that our results may not generalize to other schizophrenia patients. Third, patients with schizophrenia did exhibit a slight, non-significant impairment in their ability to detect flickers. It is therefore possible that the results that we report reflect a lack of attentional engagement during the task. However, the presence of a clear VEF in each MEG – as well as concurrent monitoring of eye-gaze – suggests that patients with schizophrenia did indeed engage with the task. Moreover, there was no relationship between DCM parameters and sensitivity to detect flickers. Fourth, all but one of the schizophrenia patients were taking antipsychotic medication, raising the possibility that group differences were either caused by or even masked by medication. A recent paper using DCM supports the latter possibility (71). In that study, both individuals at risk for psychosis and in a first-episode exhibited FPN dysconnectivity (assessed using fMRI); but this was normalized by antipsychotic medication. However, in the present study we did not detect any correlation between chlorpromazine equivalent dosage and behavioral performance or DCM coupling estimates. Finally, it is important to note that these results require replication, especially as the results for the between-group comparison in sensor-space did survive correction for multiple comparisons.

In summary, these data support the notion of FPN dysconnectivity in schizophrenia. This exists despite patients with schizophrenia being able to engage in the task and perform at a high standard on average. This dysconnectivity was mainly reflected in a reduction in top-down connectivity between the right IPS and TPJ in patients when processing attended stimuli. This reduction was associated with low premorbid IQ in the schizophrenia group, and may indicate aberrant integration between the dorsal and ventral components of the FPN. Future work should investigate the association between FPN dysconnection and impairment on specific neurocognitive measures, and assess the impact of antipsychotic medication.

## Conflict of Interest Statement

The authors declare that the research was conducted in the absence of any commercial or financial relationships that could be construed as a potential conflict of interest.
